# Effectiveness of blended learning versus lectures alone on ECG analysis and interpretation by medical students

**DOI:** 10.1186/s12909-020-02403-y

**Published:** 2020-12-03

**Authors:** Charle André Viljoen, Rob Scott Millar, Kathryn Manning, Vanessa Celeste Burch

**Affiliations:** 1 Division of Cardiology, Groote Schuur Hospital, Faculty of Health Sciences, University of Cape Town, Observatory, Cape Town, 7925 South Africa; 2 Department of Medicine, Groote Schuur Hospital, Faculty of Health Sciences, University of Cape Town, Observatory, Cape Town, 7925 South Africa; 3 Hatter Institute for Cardiovascular Research in Africa, Faculty of Health Sciences, University of Cape Town, Observatory, Cape Town, 7925 South Africa

**Keywords:** Blended learning, Electrocardiography, Medical students

## Abstract

**Background:**

Most medical students lack confidence and are unable to accurately interpret ECGs. Thus, better methods of ECG instruction are being sought. Current literature indicates that the use of e-learning for ECG analysis and interpretation skills (ECG competence) is not superior to lecture-based teaching. We aimed to assess whether blended learning (lectures supplemented with the use of a web application) resulted in better acquisition and retention of ECG competence in medical students, compared to conventional teaching (lectures alone).

**Methods:**

Two cohorts of fourth-year medical students were studied prospectively. The conventional teaching cohort (*n* = 67) attended 4 hours of interactive lectures, covering the basic principles of Electrocardiography, waveform abnormalities and arrhythmias. In addition to attending the same lectures, the blended learning cohort (*n* = 64) used a web application that facilitated deliberate practice of systematic ECG analysis and interpretation, with immediate feedback. All participants completed three tests: pre-intervention (assessing baseline ECG competence at start of clinical clerkship), immediate post-intervention (assessing acquisition of ECG competence at end of six-week clinical clerkship) and delayed post-intervention (assessing retention of ECG competence 6 months after clinical clerkship, without any further ECG training). Diagnostic accuracy and uncertainty were assessed in each test.

**Results:**

The pre-intervention test scores were similar for blended learning and conventional teaching cohorts (mean 31.02 ± 13.19% versus 31.23 ± 11.52% respectively, *p* = 0.917). While all students demonstrated meaningful improvement in ECG competence after teaching, blended learning was associated with significantly better scores, compared to conventional teaching, in immediate (75.27 ± 16.22% vs 50.27 ± 17.10%, *p* <  0.001; Cohen’s *d* = 1.58), and delayed post-intervention tests (57.70 ± 18.54% vs 37.63 ± 16.35%, *p* <  0.001; Cohen’s *d* = 1.25). Although diagnostic uncertainty decreased after ECG training in both cohorts, blended learning was associated with better confidence in ECG analysis and interpretation.

**Conclusion:**

Blended learning achieved significantly better levels of ECG competence and confidence amongst medical students than conventional ECG teaching did. Although medical students underwent significant attrition of ECG competence without ongoing training, blended learning also resulted in better retention of ECG competence than conventional teaching. Web applications encouraging a stepwise approach to ECG analysis and enabling deliberate practice with feedback may, therefore, be a useful adjunct to lectures for teaching Electrocardiography.

**Supplementary Information:**

The online version contains supplementary material available at 10.1186/s12909-020-02403-y.

## Background

The incorrect interpretation of an electrocardiogram (ECG) may lead to inappropriate clinical decisions with adverse outcomes [1, 2]. Although computerised ECG diagnostic algorithms are available, these are frequently not accurate and clinicians should therefore not rely on these automated ECG interpretations [3–6]. ECG interpretation is thus an essential learning outcome in undergraduate medical curricula [7, 8]. The concern is that medical students around the world lack competence and confidence in ECG analysis and interpretation [9–14]. For this reason, it is important to review the way that Electrocardiography has been conventionally taught.

With the widespread availability of computers and the Internet, contemporary health professions’ education increasingly uses e-learning to supplement classroom-based teaching such as lectures [15, 16]. In Electrocardiography, computer-assisted instruction (CAI) dates back to the 1960’s when analogue computers were used to teach ECGs to medical students [17]. However, since the turn of the millennium, web-based learning has been increasingly used as a method of ECG instruction [18]. Recent work has shown that an online programme facilitating repeated ECG interpretation with deliberate practice and feedback enhanced learning [19]. Although web-based learning has previously been shown to be at least as effective as conventional methods of instruction in health sciences [20], e-learning on its own has not conclusively been shown to be more effective than lecture-based training for the acquisition of ECG analysis and interpretation skills (hereafter referred to as ECG competence) [18]. A sub-analysis of this meta-analysis showed that blended learning (face-to-face lectures complemented by e-learning) [21] had a positive impact on the acquisition of ECG competence. However, the effectiveness of blended learning on the retention of ECG competence remains unknown [18].

**Fig. 1:**
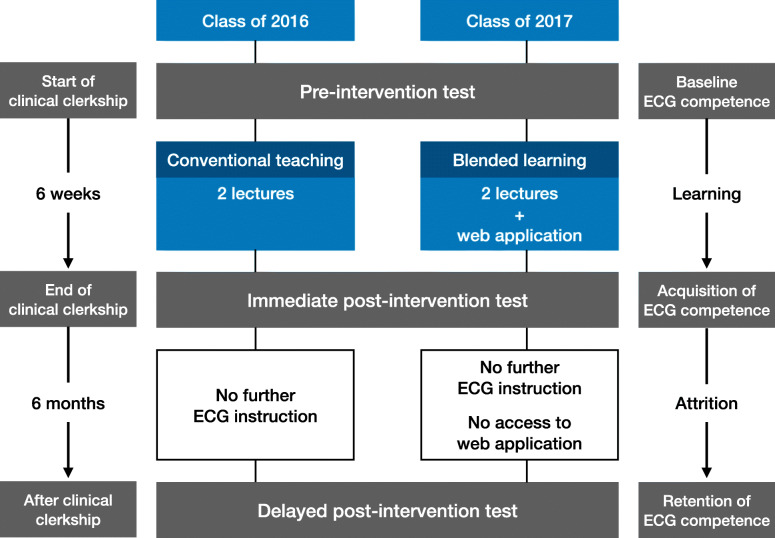
Outline of study design

The aim of our study was therefore to compare the effectiveness of blended learning (combination of face-to-face lectures and e-learning) to conventional ECG teaching (face-to-face lectures only) on the acquisition and retention of ECG competence of medical students (Fig. 1).

## Methods

### Study design and participants

This prospective study, conducted from January 2016 to November 2017, included two cohorts of fourth year medical students at the University of Cape Town, South Africa. Medical students were recruited during their Internal Medicine clinical clerkship, during which Electrocardiography is traditionally taught. Students recruited in 2016 formed the conventional teaching cohort, whereas students from 2017 formed the blended learning cohort.

### Method of ECG instruction

At the University of Cape Town, medical students are introduced to the basic principles of Electrocardiography during their third year of study, when they attend a series of lectures introducing rhythm and waveform abnormalities. Therefore, all participants in this study had prior exposure to ECG teaching in the preceding academic year. Training in Electrocardiography continues during the fourth-year Internal Medicine clinical clerkship in the form of lectures. Over and above lectures, the clinical clerkship also requires of students to acquire and analyse ECGs on the patients that they see. They present patients and their ECGs to senior clinicians on ward rounds. Although a year apart, both cohorts completed the same Internal Medicine clinical clerkship, with the same clinicians, and the same learning requirements and opportunities, both during lectures and on ward rounds. There is no formal ECG training after the Internal Medicine clinical clerkship. The other fourth-year clerkships at our institution comprise Obstetrics, Neonatology, Psychiatry and Public Health.

During the study period, participants attended two lectures (of 120 min each) at the end of the second and fourth week of the Internal Medicine Clerkship respectively. The lectures revisit the basic principles of ECG analysis (including calculation of the heart rate, measuring the different intervals and calculating the QRS axis), and then predominantly focus on waveform abnormalities (e.g. left and right atrial enlargement, left and right ventricular hypertrophy [LVH, RVH], left and right bundle branch block [LBBB, RBBB], left anterior fascicular block, Wolff-Parkinson-White [WPW] pattern, ST-segment elevation myocardial infarction [STEMI], pericarditis, hyperkalaemia, long QT syndrome) and rhythm abnormalities (e.g. sinus arrhythmia, sinus arrest with escape rhythm, first degree atrioventricular [AV] block, Mobitz I and II second degree AV block, third degree AV block, atrial fibrillation [AF] with normal and uncontrolled rates, atrial flutter, AV node re-entry tachycardia [AVNRT], ventricular tachycardia [VT] and ventricular fibrillation [VF]). The topics included in the syllabus are considered core knowledge for undergraduate ECG training at our Institution [22]. All ECGs were 12-lead ECGs, with the exception of VF for which a chest lead rhythm strip was shown. The lectures were interactive, i.e. students were asked to analyse and interpret all the ECGs shown during the Microsoft® PowerPoint® presentation, and they were encouraged to ask questions. All lectures were facilitated by the same lecturer, who used the same Microsoft® PowerPoint® presentations (demonstrating the same ECG examples and illustrations) throughout the study. Depending on the student allocations to clinical clerkships, the group sizes varied between 32 and 42 students.

Both cohorts received the same lectures. In addition, the blended learning cohort had access to a web application (ECG ONLINE, accessed at ecgonline.uct.ac.za), which facilitated ECG analysis and interpretation with feedback. Access was free, but restricted to University staff and registered students at the time of the study. Use of the web application was voluntary; its use was not a compulsory learning activity during the clerkship. Once signed into the web application, users had access to five online modules, each containing four to six ECGs to analyse. The ECGs used in the web application were different to the ECGs used during the lectures, but were of the same diagnoses discussed in class. For each of the 24 ECGs contained in the web application, the user was provided with a standardised template for online analysis, as shown in Supplementary Material 1. The template contained checkboxes for normal and abnormal parameters, as well as textboxes for interval measurements and axis calculations. For each ECG, the user selected the checkboxes that were relevant to the ECG analysis and entered the values of the interval measurements and axis calculations. The web application required that users analysed the rate and rhythm before proceeding to the detailed waveform analysis, and prior to providing their interpretation (diagnosis) of the ECG [23]. Once the process of ECG analysis and interpretation were complete and submitted, users were provided with the correct answers on the same page to facilitate comparison with the answers they had provided. For each analysed ECG, the user could also download a document with a take-home message (with text and annotated ECGs), as shown in Supplementary Material 2. There was no limit to the number of times that participants could analyse the ECGs. Students could also review their previous analyses, along with the answers of all their previously submitted ECGs. The web application monitored the number of ECGs analysed by each user.

The features offered by the web application used in this study, as well as the ECG curriculum taught online, are summarised in Table 1. These aspects were compared to undergraduate ECG teaching software that has previously been described in the literature [18, 24–33], and assessed by the modified Kirkpatrick framework [34].

**Table 1:** Comparison of the web application used in this study (ECG ONLINE) and undergraduate ECG teaching software previously described in the literature

Author	Viljoen	Akbarzadeh	Chudgar	Davies	Fent	Montassier	Nilsson	Patuwo	Rui	Sonali
Reference number	Index study	[24]	[25]	[26]	[27]	[28]	[29]	[30]	[31]	[32]
**Web application features (how ECGs were taught)**										
Online lecture / video					X				X	
Online text / images	X	X	X		X	X	X		X	?
Systematic analysis (step by step approach)	X		X							
Practice / quiz with feedback	X	X	X	X		X	X		X	
Case scenarios	X		X			X	X			
Simulation					X			X		
Online chat rooms						X				
**ECG curriculum on web application (what was taught)**										
Basic principles / pathophysiology	X	X		X	X	X	X	X	X	X
Normal ECG			X		X				X	
Normal / Abnormal rhythms	X		X		X	X	?		X	
Normal / Abnormal waveforms	X	X	X		X	X	?		X	
**Educational approach**										
Blended learning (e-learning in addition to lectures)	X		X				X		X	
Unrestricted access	X		X			X	X			
**Evaluation of web application according to the modified Kirkpatrick framework**										
Level 1: Participants’ reactions	X	X	X	X	X	X	X		X	
Level 2a: Modifications of attitudes and perceptions	X		X	X	X					
Level 2b: Acquisition of knowledge and skills	X	X	X	X	X	X	X	X	X	X
**Assessment of ECG competence**										
Immediately after educational intervention (acquisition of ECG competence)	X	X	X	X	X	X	X	X	X	X
Delayed testing after educational intervention (retention of ECG competence)	X				X					

*X = described by authors*

*? = not specifically mentioned, but implied by the text*

### Assessment of ECG competence

The study flow of participants and competence tests is outlined in Fig. 1. During the study, participants were asked to complete three 30-min competence tests. Each comprised 28 single best answer multiple-choice questions (MCQ). The first MCQ test (pre-intervention test) was written on enrolment, i.e. in the first week of the Internal Medicine clinical clerkship, prior to any ECG teaching in that academic year, to determine *baseline (pre-existing) ECG competence*. The second MCQ test (immediate post-intervention test) was written at the end of the six-week clinical clerkship, after ECG tuition (with or without access to e-learning in the blended learning and conventional teaching cohorts respectively), to assess the participants’ *acquisition of ECG competence*. The third MCQ test (delayed post-intervention test) was written 6 months later, without any further ECG training, or access to the web application, to assess the participants’ *retention of ECG competence*. The first, second and third MCQ tests respectively were the same for both cohorts, i.e. the two cohorts underwent the same assessment of baseline ECG competence, as well as acquisition and retention of ECG competence.

The three tests examined the same topics, using the same multiple-choice questions and answers, but with different exemplar ECGs in the three respective tests. Each test included three questions regarding basic ECG analysis (i.e. calculating the rate, measuring the QRS width and determining the QRS axis), as well as 25 ECG diagnoses. Of these, 12 were rhythm abnormalities (sinus arrhythmia, sinus arrest with escape rhythm, first degree AV block, Mobitz I and II second degree AV block, third degree AV block, AF with normal and uncontrolled rates, atrial flutter, AVNRT, VT and VF), and 13 were waveform abnormalities (left and right atrial enlargement, LVH, RVH, LBBB, RBBB, left anterior fascicular block, WPW pattern, anterior and inferior STEMI, pericarditis, hyperkalaemia, long QT syndrome). These conditions that were included in the MCQ tests are considered core knowledge for the undergraduate ECG training at our Institution [22]. The ECGs used in the tests were not the same as those used in class or on the web application. Two cardiologists and two specialist physicians, with a special interest in Electrocardiography, agreed that the ECGs used in the tests were unequivocal examples of the conditions and that the multiple-choice options were fair for the given ECG.

The MCQ tests were administered electronically at the University computer laboratories. They were invigilated, password-protected and could only be accessed on the day of the test. The order in which the questions were asked was randomised. For each question, there were five optional answers - four possible diagnoses (of which only one was correct), and a fifth option, i.e. “I am not sure what the answer is”. Each correct question was awarded one mark and negative marking was not applied. The answers to the questions were only made available to the students at the end of the study. The results of the MCQ tests in this study did not contribute to the participants’ course mark.

### Survey of confidence in ECG interpretation

After the immediate post-intervention test, participants completed a survey in which they were asked to rate their confidence in ECG analysis and interpretation using 5-point Likert-type questions (to which the participants could select strongly agree, agree, neutral, disagree or strongly disagree) [35, 36]. For purposes of analysis, the responses were clustered into three categories (agree, neutral and disagree).

### Determining other learning materials used during study period

After the immediate post-intervention test, participants were also asked to declare which learning materials (i.e. textbooks, class notes) they used during the study period.

### Students’ perception of lectures and web-based learning

Participants were asked to comment on what they liked and what they disliked of the lectures (both the conventional teaching and blended learning cohorts) and web application (blended learning cohort only). The feedback was received in free-text form. Two investigators (CAV, VCB) performed qualitative content analysis of the feedback from the participants. An inductive approach was used to identify themes and subthemes from the free-text comments made by the participants with regards to the lectures and web application [37, 38]. The themes and subthemes were refined through an iterative process of reviewing the participants’ responses [39]. Disagreement was resolved through discussions with a third investigator (RSM). A deductive approach was used to quantify the frequency in which the themes and subthemes emerged from the feedback on the lectures and web application [40].

### Estimated sample size needed for an adequately powered study

We estimated that a minimum sample size of 36 participants in each group would provide 80% power to detect a mean difference of 10% in the test scores after intervention between the two groups and considering an α (type 1 error) of 5%. This calculation was based on the results of previous studies that compared blended learning (lectures complemented by CAI) to lectures alone for teaching Electrocardiography [18, 25, 29, 31].

### Eligibility to be included in the study

All fourth-year medical students were invited to take part in the study; participation was voluntary. Participants were only included if they completed all three MCQ tests and the survey on ECG confidence during the study period.

### Statistical analysis

Statistical analyses were performed on anonymised data using Stata (Version 14.2, StataCorp, College Station TX, USA). The Shapiro-Wilk test was used to assess distributional normality of data [41]. Parametric data were summarised as means with standard deviations (SD), whereas median with interquartile range (IQR) were used for non-parametric data. Paired and unpaired t-tests were used to assess within-group and between-group differences in test scores respectively. Cohen’s *d* was used to determine the effect size (practical significance) of the differences in test scores, with values of 0.2, 0.5 and 0.8 indicating small, moderate and large effect sizes respectively. Categorical variables were expressed as frequencies and percentages. Chi-squared or Fisher’s exact tests were used, where applicable, to compare categorical variables. A *p* value of < 0.05 was considered statistically significant.

## Results

### Study population

All fourth-year medical students were invited to participate in the study. The conventional teaching cohort consisted of 67 of the 86 students from the 2016 class, whereas the blended learning cohort comprised 64 of 98 students from the 2017 class.

### Use of ECG learning material during the study period

**Fig. 2:**
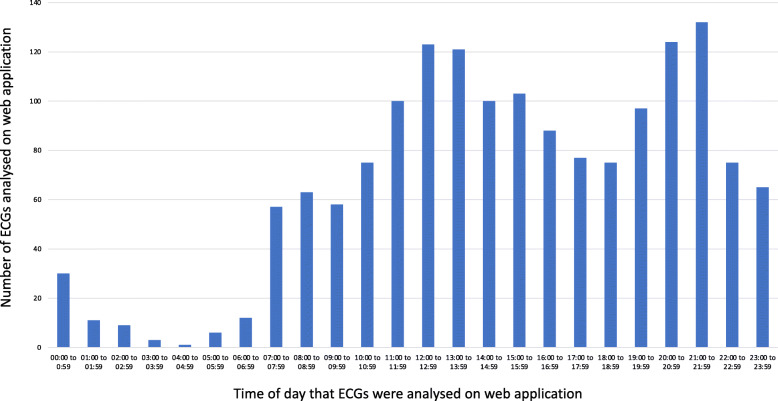
The web application was accessed throughout the day, but peaked around midday and early evening

All students in the blended learning cohort accessed the web application. Of the 24 ECGs on the web application, the median number of ECGs that were analysed and interpreted was 24 (IQR 21–24). After having analysed all the ECGs they had access to, almost two-thirds (64.2%) of the participants analysed at least one of the 24 ECGs more than once. As depicted in Fig. 2, the web application was used throughout the day, but the peak times were around midday and early evening. Those who had access to the online modules used textbooks less often than the group who attended lectures only (31.3% vs 56.1%, *p* = 0.003). However, both groups made similar use of their class notes to study ECGs (68.6% vs 71.6%, *p* = 0.717).

### Baseline ECG competence

As shown in Table 2, the cohorts exposed to blended learning or conventional teaching started with similar baseline ECG competence (mean pre-intervention test scores of 31.0% ± 13.2% and 31.2 ± 11.5% respectively, *p* = 0.917).

**Table 2:** The effect of blended learning versus conventional teaching on ECG competence

	Conventional teaching Mean % (SD)	Blended learning Mean % (SD)	***P*** value *
**Total test score (28 test items)**			
Pre-intervention test	31.2 (11.5)	31.0 (13.2)	0.917
Immediate post-intervention test	50.3 (17.1)	75.3 (16.2)	< 0.001
Delayed post-intervention test	37.6 (16.4)	57.7 (18.5)	< 0.001
**Basic analysis (3 tests items)** *†*			
Pre-intervention test	31.8 (27.5)	37.5 (25.6)	0.225
Immediate post-intervention test	57.2 (25.2)	68.6 (22.9)	0.007
Delayed post-intervention test	37.6 (16.4)	54.2 (26.9)	< 0.001
**Rhythm abnormalities (12 tests items)** *‡*			
Pre-intervention test	33.9 (15.5)	34.7 (15.9)	0.759
Immediate post-intervention test	47.3 (22.4)	73.3 (22.0)	< 0.001
Delayed post-intervention test	38.4 (19.0)	59.0 (21.5)	< 0.001
**Waveform abnormalities (13 tests items)** *§*			
Pre-intervention test	27.4 (14.8)	27.4 (17.3)	0.990
Immediate post-intervention test	51.4 (19.2)	78.6 (17.9)	< 0.001
Delayed post-intervention test	37.9 (20.3)	57.3 (22.5)	< 0.001

*SD* Standard deviation

*Unpaired T test of the difference in the mean scores between the cohorts exposed to blended learning and conventional teaching respectively

†*Calculating the QRS rate, measuring the QRS width and determining the QRS axis*

‡*Sinus arrhythmia, sinus arrest with escape rhythm, first degree AV block, Mobitz type I second degree AV block, Mobitz type II second degree AV block, third degree AV block, atrial fibrillation with uncontrolled rate, atrial fibrillation with normal rate, atrial flutter, AV node re-entry tachycardia, ventricular tachycardia and ventricular fibrillation*

§*Left anterior fascicular block, left bundle branch block, right bundle branch block, left atrial enlargement, right atrial enlargement, left ventricular hypertrophy, right ventricular hypertrophy, anterior ST-segment elevation myocardial infarction (STEMI), inferior STEMI, pericarditis, hyperkalaemia and  long QT syndrome*

### Acquisition of ECG competence

**Fig. 3:**
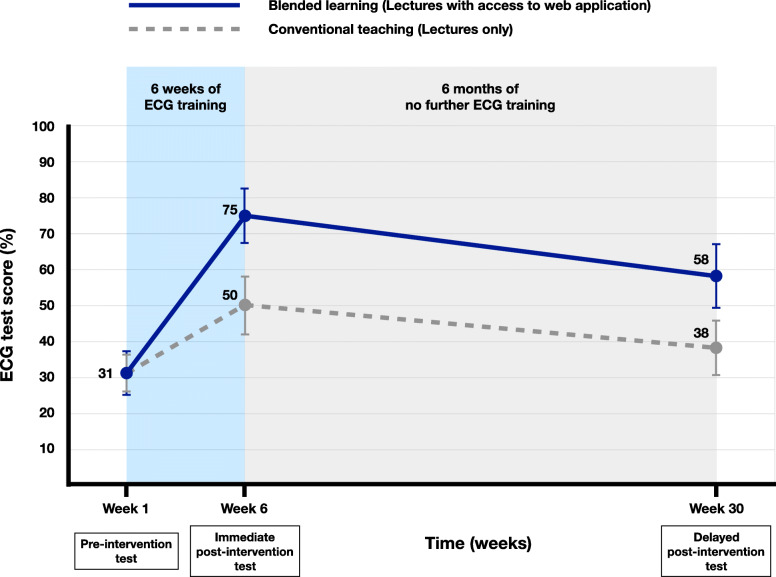
Learning and attrition with blended learning versus conventional teaching

Both cohorts showed a significant improvement in ECG competence after 6 weeks of training (Fig. 3). The conventional teaching cohort demonstrated a 1.6-fold increase in the mean test scores (31.2 ± 11.5% [pre-intervention test] to 50.3 ± 17.1%, *p* <  0.001 [immediate post-intervention test]; Cohen’s *d* = 1.3 [95% CI 0.9, 1.6]), whereas a 2.4-fold improvement in mean test scores was observed in the cohort exposed to the blended learning strategy (31.0% ± 13.2% to 75.3 ± 16.2%, *p* <  0.001; Cohen’s *d* = 3.1 [95% CI 2.6, 3.6]). The difference in acquisition of competence test scores between the two cohorts was also highly significant (Cohen’s *d* = 1.5 [95% CI 1.1, 1.9]). These test performance improvements were observed for basic analysis, as well as for the interpretation of rhythm and waveform abnormalities in the immediate post-intervention test (Table 2).

### Retention of ECG competence

After 6 months of no further ECG training, both cohorts demonstrated attrition of ECG competence (Table 2, Fig. 3). ECG competence declined significantly between the immediate and delayed post-intervention tests in the conventional teaching cohort (50.3 ± 17.1% to 37.6 ± 16.4%, *p* <  0.001, Cohen’s d = − 0.8 [95% CI -1.1, − 0.4]). Of note is that the delayed post-intervention test score in the conventional teaching cohort was similar to that of their pre-intervention test score (31.2 ± 11.5% vs 37.6 ± 16.4%, *p* <  0.001, Cohen’s *d* = 0.4 [95% CI 0.1, 0.8]). The attrition of ECG competence between the immediate and delayed post-intervention tests was also significant in the blended learning cohort (mean score of 75.3 ± 16.2% to 57.7 ± 18.5%, *p* < 0.001, Cohen’s *d* = − 1.0 [95% CI -1.4, − 0.6]). However, in this cohort of students, ECG competence at 6 months remained almost twice as much as their initial performance in the baseline test (57.7 ± 18.5% vs 31.0% ± 13.2%, *p* < 0.001, Cohen’s *d* = 1.7 [95% CI 1.3, 2.1]). Indeed, the blended learning cohort’s performance in the delayed post-intervention test was better than that achieved by the students who received conventional teaching immediately post-intervention (mean score of 57.7 ± 18.5% vs 50.3 ± 17.1%). Again, significant differences in test scores were observed for basic analysis, as well as for the interpretation of rhythm and waveform abnormalities between the two cohorts in the delayed post-intervention test.

### Decreased diagnostic uncertainty after training

As shown in Table 3, during the pre-intervention test, the “I am not sure what the answer is” option (indicating diagnostic uncertainty) was selected by the blended learning cohort as the response for 27.5% of the questions. In the same test, participants who attended conventional lectures indicated diagnostic uncertainty for 22.1% of the submitted answers. For both cohorts, there was a significant reduction in the diagnostic uncertainty in the immediate and delayed post-intervention tests. Post-intervention analysis of the responses, for which there was initial diagnostic uncertainty, translated to a significantly higher proportion of correct responses in the blended learning cohort versus the cohort that only attended lectures. This effect was still observed after 6 months.

**Table 3:** The effect of blended learning versus conventional teaching on diagnostic uncertainty and accuracy

	Conventional teaching (***n*** = 67)	Blended learning (***n*** = 64)
**Diagnostic uncertainty** ^a^		
Pre-intervention test	415 / 1876 (22.1%)	492 / 1792 (27.5%)
Immediate post-intervention test	133 / 1876 (7.1%)	69 / 1792 (3.9%)
Delayed post-intervention test	121 / 1876 (6.4%)	120 / 1792 (6.7%)
**Diagnostic accuracy for items for which there was diagnostic uncertainty in the pre-intervention test**		
Immediate post-intervention test	168 / 415 (40.5%) ^b^	360 / 492 (73.2%) ^c^
Delayed post-intervention test	113 / 415 (27.2%) ^b^	251 / 492 (51.0%) ^c^

^a^*Diagnostic uncertainty was calculated as the percentage of “I do not know the answer” responses that were selected by each cohort in the 28 item ECG competence tests*

^b^*Based on the 415 responses for which participants in the conventional teaching cohort (N = 67) selected “I do not know the answer” in the pre-intervention ECG competence test*

^c^*Based on the 492 responses for which participants in the blended learning cohort (N = 64) selected “I do not know the answer” in the pre-intervention ECG competence test*

### Students’ confidence and competence at ECG analysis and interpretation

A comparison of participants’ perceptions of their confidence and their measured competence in ECG analysis and interpretation, is shown in Table 4. Students exposed to blended learning reported less difficulty in ECG analysis and interpretation and performed significantly better in the immediate post-intervention test. Sub-group analyses showed that students who undertook blended learning demonstrated high levels of confidence and competence in rhythm and waveform analysis. While students who attended conventional teaching reported high levels of confidence in rhythm analysis, this was not associated with high levels of diagnostic accuracy of arrhythmias. These students did, however, lack confidence in waveform analysis, which was mirrored by significantly poorer performance compared to students exposed to blended learning.

**Table 4:** The effect of blended learning versus conventional teaching on confidence and competence in ECG analysis and interpretation

Confidence				Competence			
Statements used to express confidence	Conventional teaching (***n*** = 67)	Blended learning (***n*** = 64)	***P*** value	Test items used to measure competence	Conventional teaching (***n*** = 67)	Blended learning (***n*** = 64)	***P*** value
**Overall ECG analysis and interpretation**							
“I find ECG analysis and interpretation difficult”	53.7%	18.8%	< 0.001	Basic analysis (QRS rate, width and axis) and rhythm and waveform abnormalities ^a^	50.3%	75.2%	< 0.001
**Sub-analysis of rhythm abnormalities**							
“I am confident in analysing an ECG with bradycardia”	91.0%	93.8%	0.560	Sinus arrest with escape rhythm, Mobitz type I and II second degree AV block, third degree AV block, hyperkalaemia	41.2%	75.6%	< 0.001
“I am confident in analysing an ECG with tachycardia”	89.6%	84.4%	0.378	Atrial fibrillation with uncontrolled rate, atrial flutter, AV node re-entry tachycardia, ventricular tachycardia	45.8%	57.3%	0.021
“I am confident in analysing an ECG with an irregular rhythm”	56.7%	62.5%	0.500	Sinus arrhythmia, Mobitz type I and II second degree AV block, atrial fibrillation with controlled and uncontrolled rate	50.4%	77.3%	< 0.001
**Sub-analysis of waveform abnormalities**							
“I am confident in analysing the P wave”	64.1%	93.8%	< 0.001	Left and right atrial enlargement	58.2%	84.4%	< 0.001
“I am confident in analysing the PR interval”	49.3%	64.1%	0.087	First degree AV block, Mobitz type I second degree AV block, third degree AV block	44.8%	81.3%	< 0.001
“I am confident in analysing the QRS morphology”	9.0%	56.3%	< 0.001	Left and right bundle branch block, Wolff-Parkinson-White pattern, left and right ventricular hypertrophy	53.2%	77.1%	< 0.001
“I am confident in analysing the ST segment”	41.8%	67.2%	0.004	Anterior and inferior ST-segment elevation myocardial infarction (STEMI), pericarditis	46.2%	78.6%	< 0.001

^a^*Sinus arrhythmia, sinus arrest with escape rhythm, first degree atrioventricular (AV) block, Mobitz I and II second degree AV block, third degree AV block, atrial fibrillation with controlled and uncontrolled rates, atrial flutter, AV node re-entry tachycardia, ventricular tachycardia and ventricular fibrillation, left and right atrial enlargement, left and right ventricular hypertrophy, left and right bundle branch block, left anterior fascicular block, Wolff-Parkinson-White pattern, anterior and inferior ST-segment elevation myocardial infarction (STEMI), pericarditis, hyperkalaemia, long QT syndrome*

### Students’ perception of ECG lectures

As summarised in Table 5, students from both the conventional teaching and blended learning cohorts found the lectures to be interactive and contextualised. Overall, students complimented the systematic, stepwise approach to ECG analysis and interpretation that was taught during the lectures. They liked that difficult concepts were simplified and that they were taught to understand mechanisms causing waveform and rhythm abnormalities, instead of merely memorising patterns. Whereas some students felt that there was insight to their level of understanding, others reported that the lecturer did not gauge whether they understood concepts in class or not. Although both cohorts liked the practice of ECG analysis under supervision of a lecturer, the conventional cohort in particular pointed out that there was not enough opportunity to practice in class. Those from the blended learning cohort reported that the combination of lectures and the web application was beneficial for their learning, because they could apply their knowledge. However, the students often appeared overwhelmed by the lectures, saying that there was “*too much information covered in one go*”. They did not appreciate late afternoon lectures or attending ECG lectures only midway into the clinical clerkship. The blended learning cohort, in particular, criticised the lectures as being too long and covering too much content. However, the cohort who only attended lectures commented more often that the lectures were too few and too sporadic. The cohort that received conventional teaching reported that the lectures consisted of too many students, there was a lack of opportunity to practise ECG analysis in class and students were afraid to participate in front of their peers (Table 6).

**Table 5:** Themes and subthemes of what participants liked about the lectures

Theme	Subtheme	Number of mentions		Example of feedback
		CT	BL	
Method of instruction	Systematic / stepwise approach	14	19	“Breakdown of ECGs such that there is a step by step approach” “Teaching was delivered in a systematic way that is easy to reproduce and most of the time leads you to the correct diagnosis”
	Interactive	12	9	“Able to ask questions and receive an answer” “I was also afforded an opportunity to ask questions if I didn’t understand”
	Supervised practice	3	2	“We got to participate in interpreting ECG with the supervision of a skilled clinician”
	Well-paced delivery	2	3	“Taught at a pace that I could follow”
	Simplifying concepts	13	10	“They made hard concepts more simple”
	Taught to understand mechanisms instead of memorising patterns	1	6	“The explanations of why patterns/ changes arise in certain conditions helped me to remember them” “If I understand how the condition works, I find that I am able to retain the information”
	Visual orientation	0	3	“I liked that it was very visual”
	Contextualised	3	3	“Use of real life ECGs as examples”
	Scaffolded learning	4	0	“It allowed me to build on my previous knowledge of ECG’s”
	Blended learning	N/A	18	“I enjoyed the combination of the online component to the ECG lecture teaching as I got to apply my knowledg.”
Lecturer attributes	Insight to level of student understanding	4	4	“It seemed like he understood what we could possibly misunderstand and catered for it in his explanations”
	Enthusiasm	2	3	“It was enthusiastically taught”
	Skilled teacher	3	1	“Great presenting and explanation”
	Engagement	0	2	“I enjoyed the way … teaches and his willingness to explain to students”
Learning environment	Safe learning space	1	1	“The lecturers were conducted in a student friendly manner and created a safe learning space”
	Clinical application	0	1	“I enjoyed being able to actually interpret ECGs and use them as a tool to diagnose patients”

*BL* Blended learning*; CT* Conventional teaching*; N/A* Not applicable

**Table 6:** Themes and subthemes of what participants disliked about the lectures

Theme	Subtheme	Number of mentions		Example of feedback
		CT	BL	
Lecture time	Too long	5	14	“Lectures were too long. They should have been broken up into 3 or 4 shorter sessions”
	Too short	15	6	“I did not like the fact that we had to learn such a crucial skill in such a short amount of time”
	Too few	10	5	“Sporadic”, “Too few”
	End of day	2	4	“Time slots in afternoon for ECG teaching can be tiring and I tended to lose focus at times during the teaching”
	Too late in clerkship	0	4	“I would have loved to have had [ECG teaching] earlier during the rotation”
Method of instruction	Content overload	5	8	“Too much information was covered in one go, therefore making it difficult to digest the information given”
	Lack of opportunity to practice in class	8	1	“The information was not retained because we couldn’t practice”
	Focus on summative assessment	1	0	“ECG teaching at the moment is very much for exam and test purposes”
	Material not available for preparation	2	1	“Although it would have been more helpful if we had access to the notes before and after in order to read up beforehand”
	Large group instruction	3	0	“There were too many of us present”
Lecturer attributes	Lecturer failed to gauge if students understood content	3	0	“Probing for understanding was not done in class”
Student attributes	Afraid of participation in front of peers	4	0	“Sometimes you are scared to ask questions because you don’t know how to phrase your confusion”
	Attrition of knowledge	4	0	“At the time, they made sense, but I forgot many things”

*BL* Blended learning*; CT* Conventional teaching*; N/A* Not applicable

### Students’ perception of online ECG learning

Tables 7 and 8 respectively depict what the blended learning cohort liked and disliked about the web application. These students commented favourably on the fact that the web application allowed for practice and revision of ECG analysis and interpretation, and that it facilitated asynchronous learning (i.e. whenever or wherever convenient, at their own pace). The most important features that were positively perceived were the systematic approach that was taught online, the take-home messages that could be downloaded for every ECG that they analysed and the immediate feedback received after they analysed and interpreted the ECG. However, the web application was criticised because, although it displayed whether the analysis was correct or incorrect and provided the correct answer, it did not indicate or explain why an answer was incorrect in the case of an incorrect submission. Although some students felt that the web application exposed them to a wide variety of ECGs, others reported that they would have liked more examples of each condition. Technical aspects such as web page layout and the intermittent ‘bugs and glitches’ were also criticised.

**Table 7:** Themes and subthemes of what participants liked about the web application

Theme	Subtheme	Number of mentions	Example of feedback
Method of instruction	Systematic / stepwise approach	30	“It helped me develop a method of approaching ECGs more systematically” “The fact that it takes you through an ECG in a step wise process - it teaches you a method”
	Deliberate practice	5	“it is possible to practice lots of ECG interpretations”
	Immediate feedback	18	“I loved … the fact that you get immediate feedback on answers” “The best part was having such immediate feedback … I learnt from my mistakes straight away without wondering where I went wrong or forgetting to follow up with a lecturer”
	Downloadable notes on ECGs	24	“The take home message diagrams and pictures were so useful in understanding and not just rote learning information” “I liked the take home messages. They were explained in a way that encouraged thinking into the pathology behind the pattern and not just pattern recognition”
	Liberal use of examples	7	“It has taught me a lot of things about different ECGs” “The wide variety of ECGs”
	Revision	3	“Being able to review ECGs you have previously analysed”
Asynchronous learning opportunities	Learning at own pace	4	“I could learn on my own at my own pace”
	Accessible where convenient	3	“Being able to access the tool off-campus”
	Accessible when convenient	2	“It is something that you can access at anytime”
Features of web application	User friendly	8	“Well-structured and easy to follow”
	Web page layout	2	“I enjoyed the layout of the ECG online website”
	Zoom function	1	“The ability to zoom easily on ECGs”

**Table 8:** Themes and subthemes of what participants disliked about the web application

Theme	Subtheme	Number of mentions	Example of feedback
Method of instruction	ECG analysis too detailed	9	“Too many options to choose from. Overwhelming”
	ECG analysis laborious	6	“It took a long time to complete one ECG”
	Inadequate feedback	7	“I did not like the fact that, the feedback only showed the correct answers and there was no explanation on how to get the answers”
	No point of reference	2	“There was no place on the website that you could go back and look if you did not know what the morphology looked like”
	Limited number of examples	2	“I wish there were more examples”
Features of web application	Bugs and glitches	10	“There were some glitches when it wouldn’t work”
	Web page layout	13	“I think the layout can be improved a bit more”

## Discussion

This study evaluated the effectiveness of using a blended learning strategy (i.e. conventional lectures supplemented with the use of a web application) and compared results to conventional teaching (i.e. lectures alone) of ECG analysis and interpretation skills in fourth year medical students. We found that blended learning was associated with greater confidence and competence in ECG analysis and interpretation skills, immediately after the educational intervention. These gains were evident in basic ECG analysis (i.e. calculating the heart rate, QRS width and axis), as well as in the interpretation of rhythm and waveform abnormalities. Whilst there was attrition of ECG competence in both groups after 6 months of no further ECG teaching, those exposed to blended learning retained significantly more ECG analysis and interpretation skills than their counterparts who only attended lectures. Although diagnostic uncertainty decreased with both blended learning and conventional ECG teaching, those who engaged in blended learning activities had significantly better diagnostic accuracy in the immediate and delayed post-intervention tests for topics for which they initially reported diagnostic uncertainty.

There are various possible reasons for the superior gains in ECG competence with a blended learning strategy. First and foremost, blended learning potentially increases the time that students spend on ECG learning. Many have argued that current undergraduate medical training does not allow sufficient time for this activity [7, 8, 42, 43]. In our study, students reported that they found lectures to be too short and sporadic. This was especially true for those from the conventional teaching cohort. In this regard, a blended learning strategy offers the benefit of additional training by means of a web application, without increasing face-to-face ECG tuition time [15, 28]. The benefit of supplementing lectures with e-learning had been shown to be most significant when students had unrestricted access to computer-assisted ECG instruction [18].

As shown in this study, an important benefit of e-learning is that it allows for asynchronous learning, because students can study the online material wherever and whenever convenient, in addition to attending face-to-face teaching [9, 28, 44, 45]. We also showed that ECG learning on the web application took place throughout the day, but peaked at midday and early evening, at which time students do not have lectures. Asynchronous e-learning supports the self-directed learning theory in which learners self-regulate their learning by planning and monitoring their learning [46–49]. Self-directed learning allows for repetitive practice and focused revision of learning material [19, 50–52], which has been shown to be of benefit. This was true in our study, as participants commented that both lectures and web application taught a systematic approach to ECG analysis and interpretation, but that the online platform allowed them the opportunity to practise and consolidate these diagnostic approaches through repetition. With self-directed and asynchronous e-learning, students can adjust the pace of their learning and spend as much time as they need to assimilate new knowledge [24, 25, 53], which is a major advantage of blended learning over conventional classroom ECG teaching. This was consistent with the feedback from the participants in this study, as the lectures were criticised for being too rushed and covering too much content in too little time, whereas with the web application they could study at their own pace.

In our study, students who undertook blended learning had the opportunity to practise ECG analysis online and receive immediate feedback. As one participant pointed out “*The best part was having such immediate feedback … I learnt from my mistakes straight away without wondering where I went wrong or forgetting to follow up with a lecturer*”. These self-administered online quizzes filled the gap of limited opportunity for practice and feedback during lectures, especially in the large group setting [42, 54]. Indeed, those who only attended lectures pointed out the lack of opportunity to practise ECG analysis in class more often. This is an important drawback of large group teaching [55]. Deliberate practice with feedback, which underpins reflective models of learning, has previously been shown to enhance learning [52, 56, 57], and improve the retention of knowledge [58, 59]. This is also true of the initial acquisition of ECG competence [18, 25, 28, 29, 60]. The findings of our study further support these observations in the literature. In addition, we show that blended learning using a web application providing immediate feedback was also associated with better retention of ECG competence 6 months after the learning activities. This has not previously been reported [18].

Our study confirms the ‘learning and forgetting curve’ described in the literature, where competence increases with training, but declines without ongoing teaching [61] and testing [62]. In this study, all students experienced attrition of ECG competence in the absence of ongoing ECG training. The attrition rate was the same over time amongst those who engaged in blended learning and those who attended lectures during their clerkship. After 6 months of no further ECG training, both cohorts retained 75% of the ECG competence gained by the respective educational interventions. However, the retention of ECG analysis and interpretation skills was significantly better in the blended learning group. This could potentially be explained by the higher level of ECG competency initially achieved by this group during the clinical clerkship.

In other domains of medicine, such as dermatology and radiology, where diagnosis also depends on a visual analysis, it has been shown that diagnostic uncertainty decreases with experience [63–66]. Our work confirms that this is true for Electrocardiography as well, whether conventional or blended learning strategies are used. However, students who engaged in blended ECG learning activities achieved better diagnostic accuracy for topics for which there was initial diagnostic uncertainty.

In addition to the lack of ECG competence, undergraduate and postgraduate students also lack confidence in ECG analysis and interpretation, which improves with training [14]. In this study we observed that medical students exposed to blended learning were more confident in ECG analysis and interpretation at the end of their clinical clerkship. This confidence matched their competence in basic ECG analysis and the interpretation of abnormal rhythms and waveforms. However, for students who were exposed to conventional ECG teaching alone, there was a dissociation between their self-reported confidence and competence in rhythm analysis.

The development of web-based learning material has the potential of being expensive and time-consuming for both lecturer and student. Although the creation of educational content requires the experience of the medical educator, the creation and maintenance of online platforms on which the educational content is ultimately hosted requires information technology (IT) skill and expertise, which might not necessarily be available at all teaching institutions [45, 67]. The implication is therefore that funding should be sought for the development and hosting of online material, which may be an additional burden to Faculty budgets. Although ECG ONLINE was offered to our students at no cost, the expense of development and hosting of the web application to date is estimated at $30,000. To make online learning financially sustainable, students are often required to pay subscriptions to access web applications. The creation of new material also requires the time of lecturers, who are often clinicians with busy practices. However, once created and accessible to students, it could offer unlimited contact time with online material. The deliberate practice with feedback on web applications is not limited to the time and availability of the lecturer. Lecturers should, however, recognise the danger of curricular overload [22, 68], and consider whether their students’ studying schedules would allow for additional learning material.

Our research has practice implications. On the basis of our study, we have implemented blended learning using a web application that facilitates deliberate practice of ECG analysis and interpretation with feedback. Whether this strategy will benefit graduating medical students and ultimately improve patient care remains to be studied. However, an additional challenge for educators will be to further improve retention of ECG competence through continuous exposure in a longitudinal curriculum [62].

### Study limitations

In a study with different cohorts, there is a possibility of performance bias, i.e. exposure to factors other than the educational intervention that may have influenced the outcomes among the different groups. Although the two cohorts reviewed ECGs with the same senior clinicians on ward rounds, it is impossible to control teaching opportunities during a clinical clerkship.

We acknowledge different participation rates in the two cohorts. This is most likely due to a change in the order of the fourth-year clinical clerkships at the University during the study period. Participants in the blended learning cohort had to travel from another hospital to take part in the retention of knowledge tests, whereas the conventional teaching cohort was already on the same campus as the computer laboratories on the day of the retention test. However, the number of eligible participants and the results of the pre-intervention tests did not differ between the cohorts.

It was beyond the scope of this study to evaluate how the students engaged with the online material. Such an appraisal would be important to improve future e-learning interventions and blended learning strategies, and should be studied in future.

## Conclusion

Our study found that a blended learning strategy resulted in better acquisition of ECG competence than lectures alone. As expected, diagnostic uncertainty decreased with both teaching modalities. However, blended learning was associated with better diagnostic accuracy in situations where there was initial diagnostic uncertainty. Whilst there was an attrition of ECG analysis and interpretation skills without further training, the six-month retention of ECG competence was better amongst those who were exposed to blended learning. Blended learning using a web application that facilitates deliberate practice of systematic ECG analysis with feedback may, therefore, be a useful addition to the learning toolbox for Electrocardiography training of medical students.

## Glossary terms

- **‘Blended learning’** refers to a combination of lectures and e-learning [69].
- **‘Computer-assisted instruction’** (CAI) refers to any teaching method that uses a digital platform as a self-directed learning technique [23].
- **‘E-learning’** refers to the teaching method whereby electronic technologies (such as web applications) are used to access learning material [69].
- ‘**ECG analysis**’ refers to the detailed examination of an ECG tracing, which requires the measurement of intervals and the evaluation of the rhythm and each waveform [23].
- ‘**ECG interpretation**’ refers to the conclusion reached after careful ECG analysis, that is, making a diagnosis of an arrhythmia, ischaemia and so on [23].
- ‘**ECG competence**’ refers to the ability to accurately analyse and interpret the ECG [23].
- ‘**ECG knowledge**’ refers to the understanding of ECG concepts, for example, knowing that transmural ischaemia or pericarditis can cause ST-segment elevation [23].

## Supplementary Information

**Additional file 1: Supplementary Material 1**. The user was provided with a short clinical vignette and ECG with a standardised template for online analysis.

**Additional file 2: Supplementary Material 2**. Example of a ‘take-home message’ that could be downloaded from the web application once the ECG was analysed and interpreted.

## Data Availability

The datasets used and/or analysed during the current study, are available in the “Effectiveness of blended learning versus lectures alone on ECG analysis and interpretation by medical students” repository, which could be accessed at 10.25375/uct.12931262.v1.

## Abbreviations

AF: Atrial fibrillation
AV: Atrioventricular
AVNRT: Atrioventricular nodal re-entry tachycardia
ECG: Electrocardiogram
ICTS: Information and communication technology services
IQR: Interquartile range
LBBB: Left bundle branch block
LVH: Left ventricular hypertrophy
RBBB: Right bundle branch block
RVH: Right ventricular hypertrophy
SD: Standard deviation
STEMI: ST-segment elevation myocardial infarction
UCT: University of Cape Town
VF: Ventricular fibrillation
VT: Ventricular tachycardia
WPW: Wolff Parkinson White
