# Supplementary material for: Effectiveness of blended learning versus lectures alone on ECG analysis and interpretation by medical students
Source: BMC Med Educ. 2020 Dec 3;20:488. doi: 10.1186/s12909-020-02403-y (PMC7713171; doi:10.1186/s12909-020-02403-y)
Supplement: Supplementary file 1 — Additional file 1: Supplementary Material 1. The user was provided with a short clinical vignette and ECG with a standardised template for online analysis. [file 12909_2020_2403_MOESM1_ESM.pdf]

Supplementary material 1: the user was provided with a short clinical vignette and ECG with a standardised template for online analysis

1A: the web application required a systematic approach to ECG analysis, starting with a rhythm overview

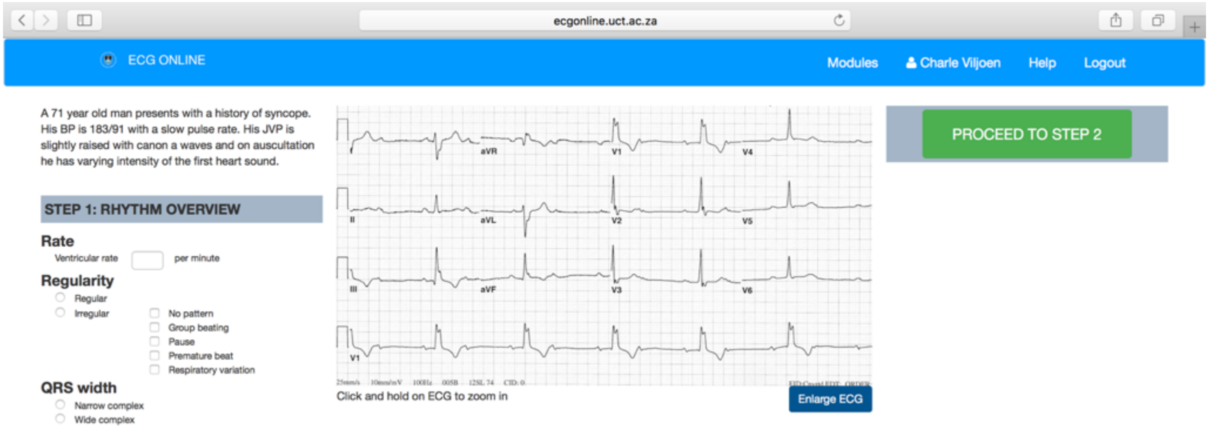

1B: the user could not proceed to the next step of their analysis without completing all the necessary fields

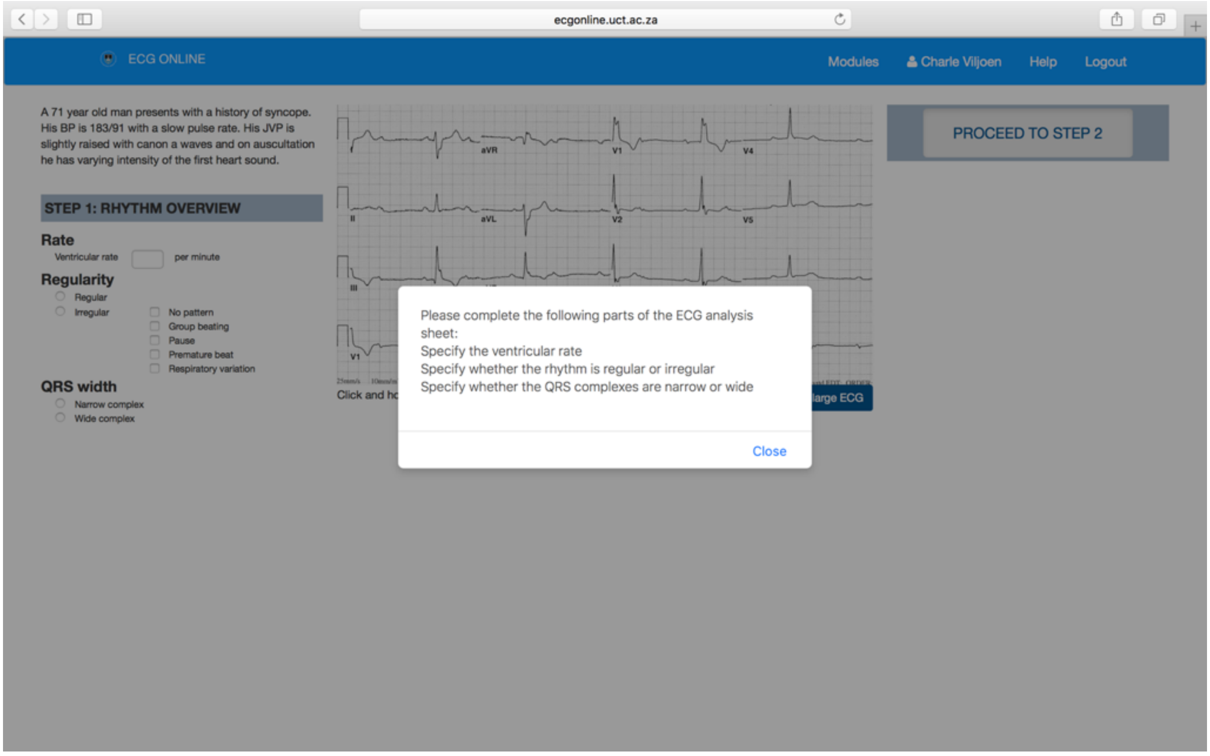

Copyright belongs to the University of Cape Town

1C: once the user completed the ECG analysis and interpretation (i.e. provided a diagnosis), they could submit their answer.

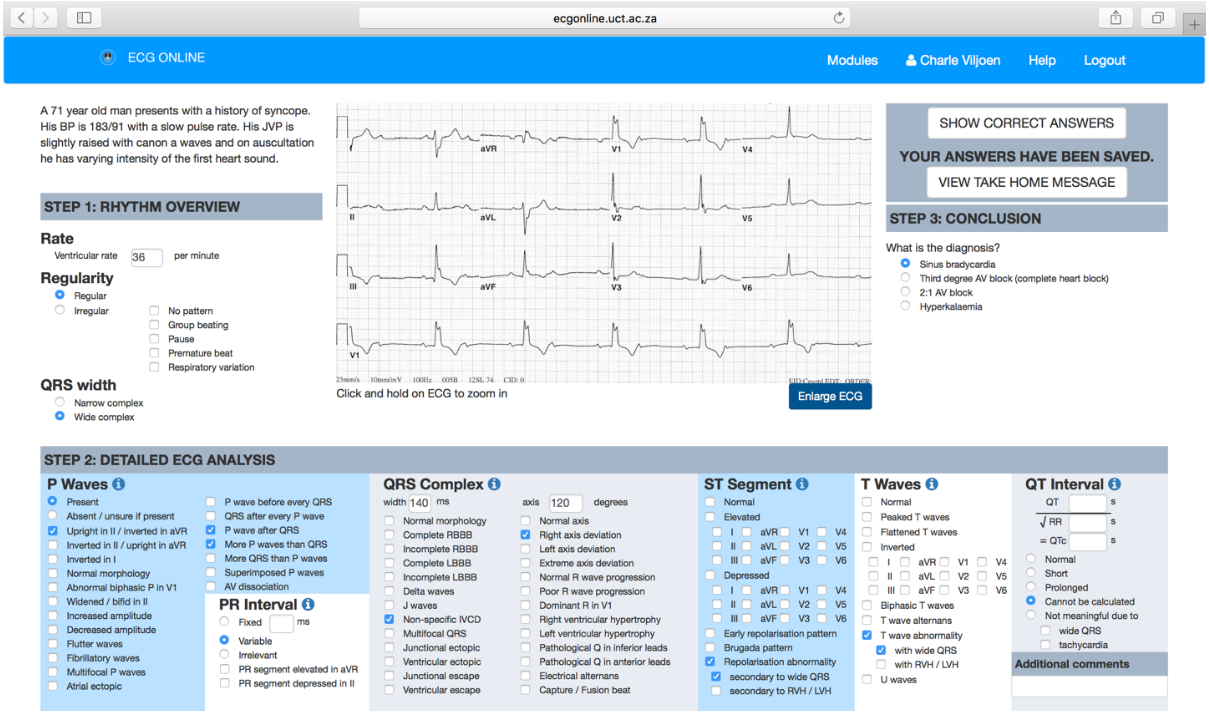

1D: the correct answers were displayed in red alongside the user's own ECG analysis and interpretation.

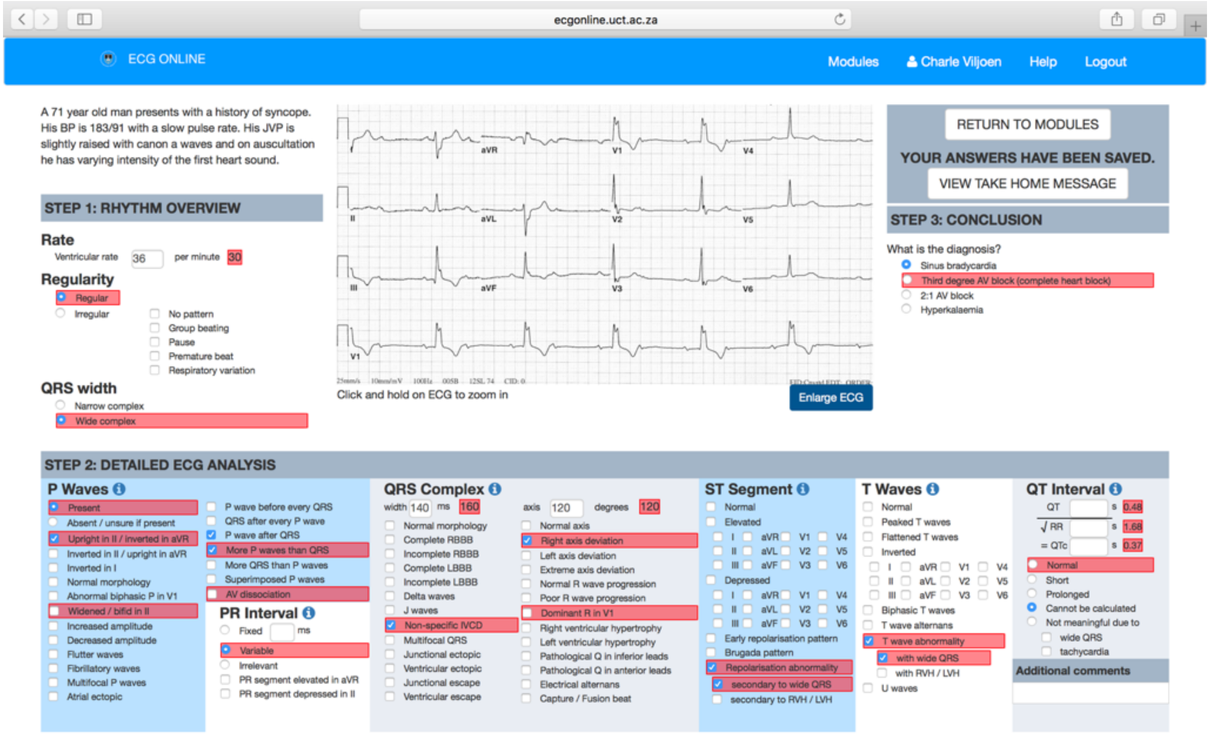

 Copyright belongs to the University of Cape Town
