# Supplementary material for: Effectiveness of blended learning versus lectures alone on ECG analysis and interpretation by medical students
Source: BMC Med Educ. 2020 Dec 3;20:488. doi: 10.1186/s12909-020-02403-y (PMC7713171; doi:10.1186/s12909-020-02403-y)

Supplementary material 2: Example of a take home message that could be downloaded from the web application once the ECG was analysed and interpreted.

**Third degree AV block (complete heart block)**

In third degree AV block there is no conduction from the atria to the ventricles.

ECG features of third degree AV block:

- Bradycardia
  - There are more P waves than QRS complexes (the atrial rate is faster than the ventricular rate)
- The P-P interval (atrial activity) is usually regular, but can be irregular
- The R-R interval (ventricular activity) is regular
- The PR interval has varying lengths. This represents AV dissociation:
  - No atrial impulses are conducted to the ventricles (there is no AV conduction)
  - The atrial and ventricular activation are independent
- The QRS complex can be narrow or wide
  - A narrow QRS complex (<120ms) represents a junctional escape rhythm
  - A wide QRS complex (>120ms) represents a ventricular escape rhythm

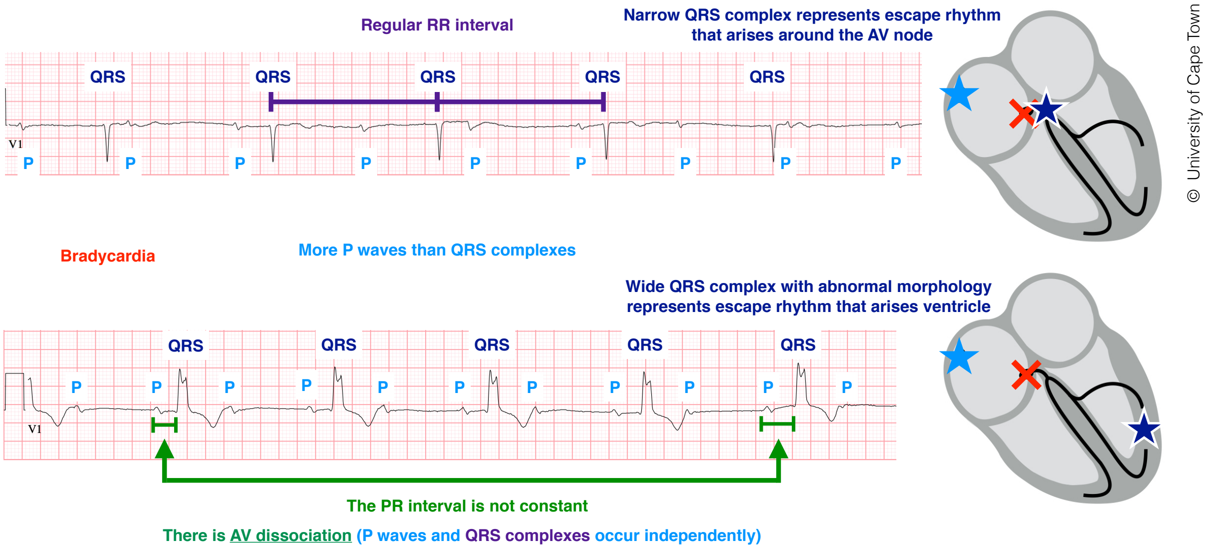

Supplement: Supplementary file 2 — Additional file 2: Supplementary Material 2. Example of a ‘take-home message’ that could be downloaded from the web application once the ECG was analysed and interpreted. [file 12909_2020_2403_MOESM2_ESM.pdf]
